# Metastatic Uterine Leiomyosarcoma Involving Bilateral Ovarian Stroma without Capsular Involvement Implies a Local Route of Hematogenous Dissemination

**DOI:** 10.1155/2015/950373

**Published:** 2015-05-24

**Authors:** Monica Dandapani, Brandon-Luke L. Seagle, Amer Abdullah, Bryce Hatfield, Robert Samuelson, Shohreh Shahabi

**Affiliations:** ^1^Department of Obstetrics, Gynecology and Reproductive Biology, Western Connecticut Health Network, 24 Hospital Avenue, Danbury, CT 06810, USA; ^2^Department of Pathology, Western Connecticut Health Network, 24 Hospital Avenue, Danbury, CT 06810, USA; ^3^Division of Gynecologic Oncology, Northwestern University Feinberg School of Medicine, 250 East Superior Street, Suite 03-2303, Chicago, IL 60611, USA

## Abstract

Uterine sarcomas spread via lymphatic and hematogenous dissemination, direct extension, or transtubal transport. Distant metastasis often involves the lungs. Ovarian metastasis is uncommon. Here we present an unusual case of a large, high-grade uLMS with metastatic disease internal to both ovaries without capsular involvement or other abdominal diseases, and discovered in a patient with distant metastases to the lungs, suggesting likely hematogenous dissemination of uLMS to the ovaries in this case. Knowledge of usual uLMS metastases may influence surgical management in select cases.

## 1. Introduction

Uterine leiomyosarcoma (uLMS) is an aggressive malignancy accounting for 1-2% of uterine malignancies and one-third of uterine sarcomas. The diagnosis of uLMS is often made incidentally during or after hysterectomy or myomectomy for presumed benign leiomyomas. Histopathologic diagnosis is determined by the presence of abundant mitoses, coagulative tumor cell necrosis, hypercellularity, and cellular atypia. Thirty percent of uLMS cases present with extrauterine disease [1].

Treatment of uLMS includes total abdominal hysterectomy (TAH) with or without bilateral salpingo-oophrectomy (BSO) [2]. Adjuvant chemotherapy or radiotherapy is offered for metastatic disease and may be considered for early stage disease. Uterine sarcomas spread via lymphatic and hematogenous dissemination, direct extension, or transtubal transport. Distant metastasis often involves the lungs. Ovarian metastasis is uncommon. Leitao et al. found that 2/71 (2.8%) uLMS cases presenting at stage I/II and 2/37 (5.4%) at stage III/IV had ovarian metastasis [3]. Similarly, Major et al. reported an adnexal metastasis incidence of 3.5% among 59 women with uLMS who underwent TAH/BSO [4]. Given the 2–5% incidence of ovarian metastasis in cases of uLMS, ovarian preservation is an option for premenopausal women with uLMS grossly confined to the uterus or for women in whom the diagnosis of uLMS was made incidentally after hysterectomy without BSO. However, unrecognized ovarian metastasis may contribute to worse outcomes. Here we present a rare case of uLMS presenting with bilateral ovarian and multiple lung metastases. Knowledge of usual uLMS metastases may influence surgical management in select cases. We also review the literature of uLMS with ovarian metastasis.

## 2. Case Presentation

**Figure 1:**
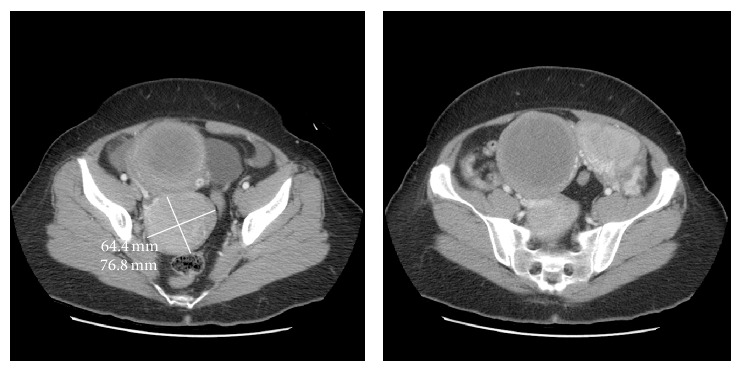
Representative computed tomography (CT) images of the abdomen and pelvis showing uLMS as a large heterogeneous uterine tumor.

A 53-year-old postmenopausal woman, gravid 4 para 3, presented to the emergency department with one week of left lower quadrant abdominal pain. She denied weight loss, fever, chills, shortness of breath, bloating, or any changes in bowel or bladder function. On physical exam, she demonstrated mild abdominal distention and mild, diffuse abdominal tenderness without guarding or rebound. Computed tomography (CT) of the chest, abdomen, and pelvis revealed bilateral adnexal masses (11.7 × 6.9 cm on the left and 6.4 × 7.7 cm on the right), an 8.9 × 8.4 cm heterogeneous lesion occupying the uterus, pelvic ascites, and extensive bilateral pulmonary nodules (Figure 1). Tumor markers, CA 125, CEA, and CA 19-9, were not elevated.

**Figure 2:**
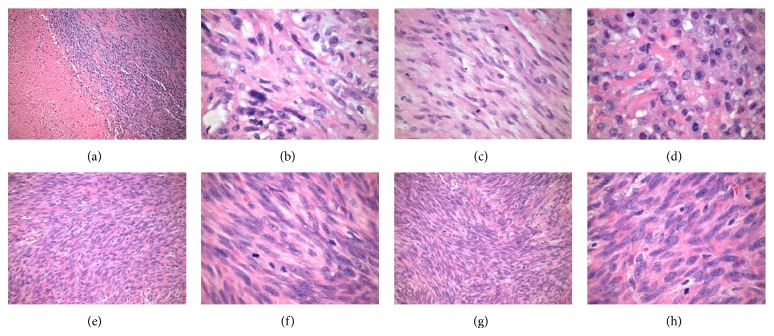
Histology. ((a)–(d)) Uterus: (a) hypercellularity with geographic necrosis (100x), (b) cellular atypia (600x), and ((c) and (d)) mitotic figures (400x). ((e)-(f)) Left Ovary. Cellular atypia and mitoses ((e) 200x and (f) 600x). ((g)-(h)) Right ovary. Cellular atypia and mitoses ((g) 200x and (h) 600x).

**Figure 3:**
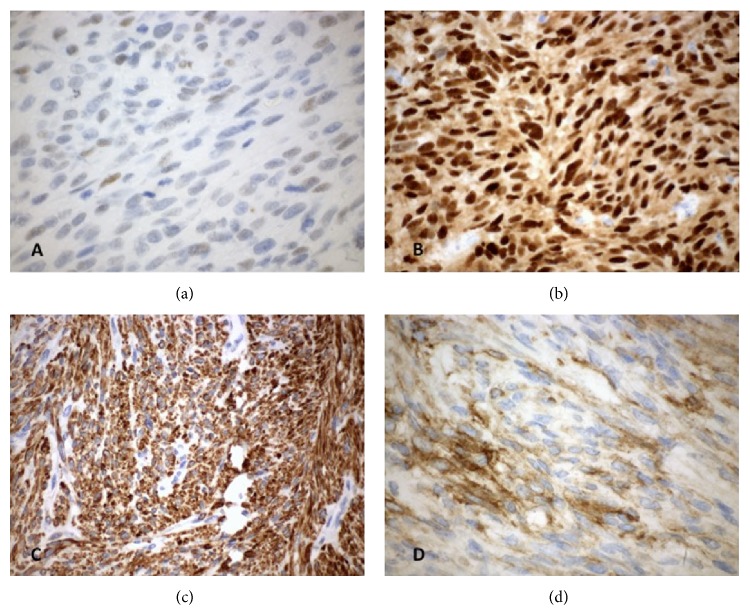
Immunohistochemistry: (a) estrogen receptor, (b) progesterone receptor, (c) desmin, and (d) CD10.

**Figure 4:**
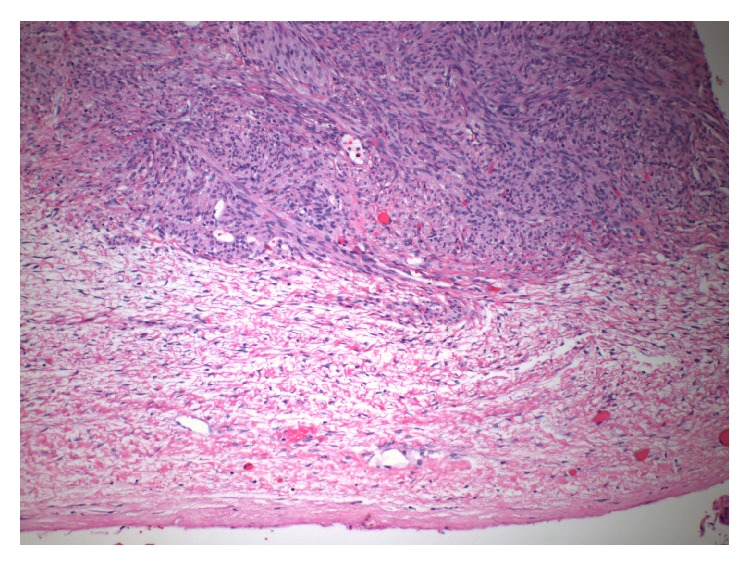
Histology: intact capsule (100x).

The patient underwent TAH/BSO. Lymphadenectomy was omitted as preoperative CT imaging and intraoperative palpation did not suggest lymphatic involvement. Final pathology demonstrated a high-grade 8.5 cm fundal uLMS and bilateral ovarian metastases. Histology included hypercellularity with tumor necrosis, cellular atypia, and mitotic figures (Figure 2). Immunohistochemistry stained strongly positive for desmin and progesterone receptors, intermediately positive for estrogen receptors, and weakly positive for CD10 (Figure 3). Both ovaries were enlarged (right 13 cm and left 9 cm), lobulated, and smooth, with intact capsules without surface involvement (Figure 4). Both fallopian tubes were patent and without disease. The omentum and appendix were without disease. Peritoneal washings were negative for malignant cells.

Given imaging evidence of pulmonary metastases, the patient was considered stage IVB and received adjuvant docetaxel and gemcitabine. Interval CT imaging showed decreased size and number of pulmonary nodules without evidence of disease progression, suggesting good clinical response. She remains asymptomatic and without evidence of disease progression 15 months after diagnosis.

## 3. Discussion

Uterine sarcomas theoretically may metastasize via direct invasion, transtubal transport, or lymphatic or hematogenous dissemination. Recently Tirumani et al. found metastasis in 81% of patients with uLMS, most commonly involving the lungs (74%), peritoneum (41%), bones (33%), or liver (27%). Ovarian metastasis is less frequent. Seven cases of uLMS with ovarian metastasis (Table 1) and six additional cases of occult ovarian metastasis are reported [3–9].

uLMS often spreads hematogenously because uLMS originates within the richly vascular myometrium and frequently presents with distant metastases. uLMS has been reported in a variety of distant locales, including the brain, gastrointestinal tract, and heart, typically with coexisting pulmonary metastases [10]. Pulmonary metastasis implies hematogenous dissemination because 100% of venous return, including uterine venous return, passes through the intricate capillary networks of the pulmonary vasculature.

Uterine malignancies that spread lymphatically often cause regional lymphadenopathy before distant metastasis, a pattern well recognized in epithelial-derived endometrial cancers. Malignant cells traveling via lymphatic networks in endometrial cancers have been mapped to pelvic and para-aortic lymph nodes, including external iliac, iliac vessel bifurcation, and aortic bifurcation nodes [11]. Lymphatic drainage also ultimately reaches the venous circulation, but this is not until the right lymphatic duct and thoracic duct reach the subclavian veins [12]. Therefore metastatic cells that have emptied into the venous circulation via lymphatic ducts could also seed distant tissue, but not without also seeding lymph nodes along the way. Leitao et al. demonstrated that 3/37 (8.1%) of uLMS patients who underwent lymph node sampling had lymphatic metastasis, and in all 3 cases, positive lymph nodes were suspiciously enlarged [3]. Lymphatic metastasis of uLMS is typically found in the presence of other extrauterine diseases [13]. Therefore, performing lymphadenectomy after incidental diagnosis of uLMS is not recommended and even if uLMS is suspected at the time of initial surgery, lymphadenectomy is typically omitted in the absence of enlarged nodes [3, 13].

Alvarado Gay and Vega Silva described a case of uLMS presenting as a 10 cm right-sided uterine mass extending into the ipsilateral fallopian tube and ovary, involving the ovarian serosa, and suggesting direct extension [5]. Another route of metastasis is transtubal transport of exfoliated cells to the ovaries. With transtubal dissemination, ovarian serosal, peritoneal, and pelvic washings may be positive for malignant cells. Bilateral ovarian metastasis of uLMS at the time of disease recurrence was described in one report [9]. A 35-year-old woman with a palpable abdominal mass who underwent hysterectomy and bilateral ovarian cystectomy was found to have a 6 cm uLMS extending to the uterine serosa. Fourteen months after initial surgery, she complained of abdominal swelling and at laparotomy was found to have bilateral ovarian metastasis with extensive disease throughout the abdomen, without evidence of distant, extra-abdominal metastasis [9]. This case likely represented direct extension of disease or transtubal transport rather than hematogenous dissemination.

It is important to also consider the possibility of synchronous development of multiple primaries. Approximately 1-2% of women with a gynecologic cancer will have two simultaneous gynecologic malignancies, the most common combination being simultaneous endometrial and ovarian malignancies [14]. Leiomyosarcoma of the uterus and leiomyosarcoma of the ovary cannot be distinguished based on histologic differences. As a result, clinical features of the tumors become more important in ruling out synchronous tumors. A large uterine tumor plus tumors in the parenchyma of both ovaries, the lack of serosal involvement, the background of other metastatic diseases, and the rarity of primary ovarian LMS are all suggestive of a uterine primary with ovarian metastases rather than synchronous development of uLMS and ovarian LMS [15, 16].

Here we present an unusual case of a large, high-grade uLMS with metastatic disease internal to both ovaries without capsular involvement or other abdominal diseases and discovered in a patient with distant metastases to the lungs, suggesting likely hematogenous dissemination of uLMS to the ovaries in this case. Blood leaving the uterus as venous return coalesces into the uterine venous plexus and then it can enter the ovarian vein, a conduit to the ovarian venous plexus [12]. Tumors cells that have invaded into the uterine venous blood could directly and immediately seed the nearby ovarian tissues and then go on to seed distant tissues such as the lung.

Given that the incidence of ovarian metastasis due to uLMS is approximately 2–5%, premenopausal women hoping to avoid surgically induced early menopause may consider ovarian preservation [3, 4]. NCCN guidelines recommend hysterectomy with the decision for BSO individualized for reproductive-age patients [2]. In premenopausal women without evidence of extrauterine disease, we advocate for unilateral oophorectomy if an ovary appears grossly unusual or enlarged at the time of initial surgery for suspected or confirmed uLMS. If extrauterine disease is suspected preoperatively, including suspicious pulmonary lesions seen on preoperative imaging studies, bilateral oophorectomy may be considered in premenopausal women with surgical decision making guided by intraoperative findings. Such patients should be counseled preoperatively regarding the possibility of bilateral oophorectomy and consequent surgical menopause. The case of bilateral ovarian metastasis of uLMS presented here is very unusual. The suggestion of hematogenous spread to the ovaries raises the possibility that early, occult ovarian metastases in cases of uLMS may be missed without routine bilateral oophorectomy. However, routinely removing normal appearing ovaries in premenopausal women with uLMS would likely result in a large number of patients suffering surgical menopause for each discovery of an occult ovarian metastasis. Given the low incidence of ovarian metastasis reported in cases of uLMS, in the absence of intraoperative or imaging findings suggesting extrauterine disease and/or ovarian pathology, routine bilateral oophorectomy for cases of suspected uLMS is unlikely to be of benefit to most women with uLMS.

## Figures and Tables

**Table 1:** Summary of the literature review of uLMS cases with ovarian metastasis.

Report	Patient age	Description	Lung metastasis?	Possible route of metastasis
Alvarado Gay and Vega Silva, 2005 [5]	33	10 cm uLMS of right lateral uterine wall involving fallopian tube, ovary, and ipsilateral parametrium plus 2 neoplasms in omentum.	no	Direct extension +/− hematogenous dissemination

Bharambe et al., 2014 [6]	65	Enlarged and lobulated uterus due to uLMS. Right ovary enlarged to 11 cm and multinodular. Left ovary unremarkable.	n/a	Direct extension, lymphatic dissemination, or hematogenous dissemination

Dai and Song, 2010 [7]	n/a	uLMS with ovarian and lymph node metastasis.	n/a	Lymphatic +/− hematogenous or direct extension

Vasiljevic et al., 2008 [8]	28	uLMS of posterior uterine wall with metastasis to capsule and cortex of right ovary. Omentum, pelvic, and para-aorta lymph nodes were negative for malignancy.	no	Direct extension

Young and Scully, 1990 [9]	35	uLMS extended from endometrium to serosa. 14 months later, ovaries enlarged and lobulated with metastatic disease plus extensive spread in abdomen.	n/a	Direct extension

Young and Scully, 1990 [9]	44	Lower uterine segment mass deemed inoperable. Seven months later debulking of uLMS involved lower uterine segment, endocervix, and paracervical soft tissue. Right ovary enlarged to 4 cm with metastatic uLMS.	n/a	Direct extension, lymphatic dissemination, or hematogenous dissemination

Young and Scully, 1990 [9]	49	uLMS creating a “rock hard” cervix, vaginal cuff, and lower uterine segment. Despite grossly unremarkable ovaries, dissection revealed one ovary with 3 discrete nodules in hilus and medulla. Parametrial and para-aortic lymph nodes metastasis was also present.	n/a	Lymphatic +/− hematogenous
